# Phosphorylation of Shrimp Tcf by a Viral Protein Kinase WSV083 Suppresses Its Antiviral Effect

**DOI:** 10.3389/fimmu.2021.698697

**Published:** 2021-08-02

**Authors:** Chuanqi Wang, Lingwei Ruan, Hong Shi, Wenyang Lin, Linmin Liu, Sujie Li

**Affiliations:** ^1^State Key Laboratory Breeding Base of Marine Genetic Resources, Key Laboratory of Marine Genetic Resources of Ministry of Natural Resources, Third Institute of Oceanography, Ministry of Natural Resources, Fujian Key Laboratory of Marine Genetic Resources, Xiamen, China; ^2^School of Life Science, Xiamen University, Xiamen, China

**Keywords:** Wnt signaling pathway, LvTcf, LvVago1, WSV083, phosphorylation, ubiquitin-proteasome pathway

## Abstract

Nuclear DNA-binding TCF proteins, which act as the main downstream effectors of Wnt signaling, are essential for the regulation of cell fate and innate immunity. However, their role during viral infection in shrimp remains unknown. Herein, we demonstrated that *Litopenaeus vannamei* TCF (LvTcf) acts independently of Lvβ-catenin to promote interferon-like protein LvVago1 production, thus mounting the response to WSSV infection. Further, we observed that WSV083, a WSSV serine/threonine protein kinase, bound to LvTcf and phosphorylated it. Phosphorylated LvTcf was then recognized and degraded *via* the ubiquitin-proteasome pathway. Moreover, mass spectrometry analyses indicated that the T39 and T104 residues of LvTcf were target sites phosphorylated by WSV083. Point mutation analyses suggested that additional sites of LvTcf may undergo phosphorylation *via* WSV083. Taken together, the current work provides valuable insights into host immunity and viral pathogenesis. LvTcf is not only a modulator of shrimp innate immunity but is also an important target for WSSV immune evasion. Thus, the current findings will help improve disease control in shrimps.

## Introduction

White spot syndrome virus (WSSV) is the only species within the Whispovirus genus of the *Nimaviridae* family (1–3). It is a large double-stranded circular DNA virus with a genome of approximately 300 kb containing 181 open reading frames (ORFs). This virus is a major crustacean pathogen, causing a cumulative mortality of up to 100% in cultured shrimp (4, 5). Due to the current lack of effective treatment, understanding the mechanisms of host immunity and host-virus interactions is of great importance for improving WSSV control.

WSSV triggers pattern recognition upon cell entry as the initial step of the innate immune response (6). Shrimp mount humoral and cellular immune responses (7) to defend against viral infection. These rely on several key cell signaling cascades, including the Toll/IMD-NF-κB and JAK/STAT pathways, among others, which transduce extracellular signals into cells and promote the expression of antimicrobial peptides or other immune effector molecules to combat WSSV infection (8–10). In its shrimp host, WSSV employs a number of mechanisms to ensure propagation. To this end, the virus hijacks host proteins to facilitate gene transcription. Shrimp NF-κB and STAT were reported to bind the promoter of the WSSV immediate early gene *ie1*, promoting its expression (11–13). Viral transcription factor IE1 then drives host JNK autophosphorylation to activate c-Jun and stimulate *ie1*, *wsv056*, *wsv249*, as well as *wsv403* expression (14). Through the cell cycle, IE1 and WSV056 competitively interact with Rb to promote the transition from G0/G1 to S phase, providing a favorable environment for viral replication (15). In addition, WSSV employs several strategies to evade host immunity. For example, viral microRNA WSSV-miR-22 restricts host STAT expression by targeting its 3′UTR, which allows for subverting the JAK/STAT-driven antiviral response (16). WSSV can also manipulate metabolic programming to induce the Warburg effect, counteracting reactive oxygen species (17–19). Moreover, WSSV regulates the degradation of host proteins *via* ubiquitination-related enzymes encoded by *wsv222*, *wsv249*, and *wsv403*, facilitating viral replication and proliferation (20–24).

Within research on the relationship between host signaling and viral infection, the Wnt pathway has attracted increasing attention. It is evolutionarily conserved in metazoan animals, regulating cell fate during embryonic development as well as in adult tissues (25–27). In addition, Wnt/β-catenin/TCF signaling participates in immune regulation. In particular, TCF-1 initiates TFH differentiation, thus promoting the B cell-mediated response to acute viral infections (28). With regard to innate immunity, β-catenin/TCF promotes the expression of type I IFN and interferon-stimulated genes to suppress viral infection (29–32). In Drosophila, the Dally-mediated Wnt signaling pathway is involved in S2 phagocytosis of WSSV (33). Our previous work showed that the expression of LvWnt5b was initially suppressed in shrimp as a proapoptotic response against WSSV infection. Moreover, Lvβ-catenin tends to translocate to the nucleus following WSSV infection where it activates the expression of several antimicrobial peptides (34, 35). However, how WSSV regulates the Wnt signaling pathway in shrimp remains unclear. Therefore, further research is necessary in order to determine the relationship between shrimp Wnt signaling and WSSV infection.

Nuclear DNA-binding TCF/LEF proteins from the high-mobility group (HMG) box family are the main downstream effectors of the Wnt pathway. In humans, the TCF/LEF family consists of four members designated TCF7, LEF1, TCF7L1, and TCF7L2 (also known as TCF1, LEF1, TCF3, and TCF4, respectively) (36). The majority of TCF isoforms possess a conserved β-catenin-binding domain at the N-terminus and a monomeric HMG domain that recognizes the Wnt response element (5’-ACATCAAAG-3’) to mediate DNA binding (37–39). Importantly, isoforms exhibit different functional properties. In this study, we found that shrimp TCF, referred to as LvTcf, plays a protective role against WSSV infection by promoting the production of shrimp interferon-like protein LvVago1 in an Lvβ-catenin-independent manner. Furthermore, we uncovered a new mechanism through which WSSV regulates the Wnt signaling pathway. WSV083, a serine/threonine protein kinase encoded by WSSV (40), was found to bind and phosphorylate LvTcf. Phosphorylated LvTcf was then degraded *via* the ubiquitin-proteasome pathway. Thus, WSV083 suppressed the antiviral effect mediated by LvTcf. The current findings highlight novel therapeutic targets for WSSV control.

## Materials and Methods

### Shrimp and Virus

*L.vannamei*, about 15-20 g, were purchased from a market in Xiamen, China, and kept in air-pumped circulating seawater for 3 days before experiments. WSSV particles (a Chinese isolated) were extracted from hemocytes of infected crayfish *Procambarus clarkii* and quantified according to Yang’s description (41, 42).

### Cell Lines, Reagents and Antibodies

High Five cells were cultured in Express Five SFM (Gibco, USA; Cat. No. 10486025) with 10% L-Glutamine (Gibco, USA; Cat. No. 25030081). S2 cells were maintained in complete Schneider’s Drosophila Medium. Complete Schneider’s Drosophila Medium was prepared as follows: Schneider’s Drosophila Medium (Gibco, USA; Cat. No. R69007) was supplemented with 10% fetal bovine serum (Gibco, USA; Cat. No. 16140071) and 1% Penicillin-Streptomycin (Gibco, USA; Cat. No. 15070063). Sf9 cells were cultured in Sf-900 III SFM (Gibco, USA; Cat. No. 10902104) with 10% fetal bovine serum and 1% Penicillin-Streptomycin. MG132 (Merck, USA; Cat. No. 474790) were used for treating cells. Calf intestinal alkaline phosphatase (CIAP, Thermo Fisher Scientific, USA; Cat. No. 18009-019) were used for dephosphorylation assay *in vitro*. Protein A-coupled Sepharose (GE Healthcare, USA; Cat. No. 17-5280-04) was used for antibody purification and immunoprecipitation. Primary Antibodies used in this study were anti-flag (Sigma, USA; Cat. No. F3165; Abcam, USA; Cat. No. ab205606), anti-V5 (Thermo Fisher Scientific, USA; Cat. No. R961-25), anti-myc (Cell Signaling Technology, USA; Cat. No. 2276S), anti-α-tubulin (Sigma, USA; Cat. No. T5168). Anti-c-myc Agarose Affinity Gel (Sigma, USA; Cat. No. A7470) and Anti-FLAG M2 Affinity Gel (Sigma, USA; Cat. No. A2220) were used for immunoprecipitation or protein purification. Secondary antibodies used in this work including Goat anti-Mouse IgG antibody (Thermo Fisher Scientific, USA; Cat. No. 31430), Goat anti-Rabbit IgG antibody (Life technologies, USA, Cat. No. A16110), FITC goat anti-mouse antibody (Life technologies, USA, Cat. No. A11029) and Rhodamine Red™-X goat anti-rabbit antibody (Life technologies, USA, Cat. No. R6394).

### RNA Extraction, cDNA Synthesis and Genomic DNA Extraction

Total RNA was extracted with TRIzol reagent (Molecular Research Center, Inc, USA; Cat. No. TR118) according to the manufacturer’s instruction. Total RNA was treated with DNaseI(Takara, Japan; Cat. No. 2270A) at 37°C for 0.5 h to remove residual genomic DNA. The first-strand cDNA was synthesized by reverse transcriptase M-MLV (Takara, Japan; Cat. No. 2641A) with Oligo(dT)_18_ primer (Thermo Fisher Scientific, USA; Cat. No. SO131). TIANGEN Marine Animal DNA Kit (TIANGEN, Beijing, China; Cat. No. GD3311-02) was used to extract the shrimp genomic DNA according to the manufacturer’s instructions.

### WSSV Challenge

Each batch of 40 healthy shrimps was injected with 1×10^5^ WSSV virions diluted in 100 μl PBS (140 mM NaCl, 3 mM KCl, 8 mM Na_2_HPO_4_, 1.5 mM KH_2_PO_4_, pH 7.4). Shrimps injected with 100 μl PBS were set as negative control. Samples of four individuals collected at different time points post injection (0, 6, 12, 24, 48 h) were used for analysis by qRT-PCR.

### RNAi Assay

We used RNAi to knock down the expression of LvTcf in order to explore its role in shrimp innate immunity. DsLvTcf and dsEGFP (control) were synthesized using the T7 RiboMAX Express RNAi System (Promega, USA; Cat. No. P1700), according to the manufacturer’s instructions. dsRNA was diluted in PBS, and 100 μL of PBS containing 20 μg dsRNA was injected into shrimps twice after 24 h. Another 12 h later, shrimp of the two groups were injected with 1×10^5^ WSSV virions. Shrimp hemocytes (n = 8) were collected at 48 h post-infection (hpi). The transcriptional levels of *LvTcf* and the number of WSSV copies were then analyzed. siRNA was used to reduce the expression of endogenous β-catenin in S2 cells. SiDmβ-catenin was synthesized by GenePharma based on the following sequences: (5′-3′) GCUUGCAAAUUCUGGCCUAT and UAGGCCAGAAUUUGCAAGCTT.

### qRT-PCR

qRT-PCR was performed using TB Green Premix Ex Taq (Cat. No. RR820) in a Rotor-Gene™ 6000 (Corbett Life Science) with the following program: 1 cycle of pre-denaturation for 1 min at 95°C, followed by 40 cycles of 95°C for 10 s, 56°C for 15 s, and 72°C for 15 s. Primers are listed in **Table S1**. We used *LvEF-1α* (GenBank accession No. GU136229) as an internal control, and each sample was analyzed in triplicate. Relative expression was determined *via* the 2^-ΔΔCt^ method. Statistical significance was set at p<0.05.

### Absolute q-PCR

We performed absolute q-PCR to monitor viral loads in shrimp. Briefly, we collected gills from shrimp at 48 hpi (n = 8 in the *LvTcf* knockdown experiment). Gill genomic DNA was extracted as described above. Primers for WSSV genomic DNA-F and WSSV genomic DNA-R (**Table S1**) were used to measure WSSV genomic copies *via* absolute q-PCR according to a previously described method (43). The WSSV copy number in 1 μg of shrimp genomic DNA was then calculated.

### Plasmid Construction

GST protein was expressed *via* the pGEX-4T-2 vector (stored in our laboratory). The ORF of WSV083 was cloned into pGEX-4T-2 to express the WSV083-GST fusion protein. The ORFs of LvTcf and Lvβ-catenin were cloned into the pIEx-4 vector (Novagen), with a c-Myc tag or FLAG tag fused to the N-terminus. The LvTcf catenin-binding domain deletion mutant (LvTcf57-556) was inserted into pIEx-4 with a c-Myc tag fused to the N-terminus. EGFP with a c-Myc, V5, or FLAG tag fused to the N-terminus was cloned into pIEx-4 vector at the same time. The ORFs of WSV056, WSV069 (IE1), WSV079, WSV100, WSV249, WSV403, wild-type (WT) WSV083, kinase domain deletion mutant WSV083DM, and kinase domain point mutant D459A WSV083PM were introduced into a pIEx-4 vector with a V5 tag fused to the N-terminus. FLAG-tagged ubiquitin was cloned into the pIEx-4 vector. The point mutations of LvTcf, including LvTcf2MuA (T39AT104A), LvTcf4MuA (T39AT104AT311AS315A), and LvTcf5MuA (T39AT104AT311AS315AS356A) were introduced into the wild-type LvTcf expression plasmid using the Mut Express^®^ II Fast Mutagenesis Kit V2 (Vazyme, Nanjing, China; Cat. No. C214). The promoters of LvVago1-5 were cloned into the pGL3-Basic vector (stored in our laboratory).

### Co-Immunoprecipitation (Co-IP) and Western Blotting Analyses

High Five cells were maintained in Express Five SFM medium with 10% L-glutamine. CellfectinTM II reagent (Thermo Fisher Scientific, USA; Cat. No. 10362100) was used for cell transfection according to the manufacturer’s instructions. High Five cells (cultured in a six-well plate) were harvested at 48 h post-transfection and lysed in Western and IP cell lysis buffer (Beyotime, Shanghai, China; Cat. No. P0013) with the addition of 1 mM phenylmethylsulfonylfluoride (PMSF; BBI Life Sciences, China, Cat. No. A610425) and a protease inhibitor cocktail (Calbiochem, USA; Cat. No. 539134) or a phosphatase inhibitor cocktail (Roche, USA; Cat. No. 04906837001) for 30 min on ice. Ten percent of the cell lysates were used for input analysis, and the rest were immunoprecipitated with an antibody affinity gel for 4 h at 4°C. Alternatively, lysates were preincubated with protein A-coupled Sepharose (GE Healthcare, USA; Cat. No. 17-5280-04) to remove nonspecific protein binding for 1 h at 4°C. Lysates were then immunoprecipitated with Sepharose and the indicated antibodies for at least 4 h. The gel or beads were washed sequentially with lysis buffer five times and boiled in 5×SDS loading buffer (Solarbio, Beijing, China; Cat. No. P1040) for western blot analysis. The protein samples were separated on SDS-PAGE gel and transferred to a nitrocellulose blotting membrane (Millipore, USA; Cat. No. IPVH00010). The membrane was blocked in 5% (w/v) skim milk in TBST (20 mM Tris-HCl, 150 mM NaCl, 0.1% Tween 20, pH 7.6) at room temperature for 1 h. The membrane was then incubated with a primary antibody for 1 h at room temperature. A horseradish peroxidase (HRP)-conjugated secondary antibody was then incubated with the membrane for 1 h at room temperature. SuperSignalTM West Pico PLUS Chemiluminescent Substrate (Thermo Fisher Scientific, USA; Cat. No. 34578) was used for signal detection.

### GST Pull-Down

A GST or GST-WSV083 expression plasmid was transformed into *Escherichia coli* BL21, and single colonies were selected on plates supplemented with ampicillin (100 μg/mL). *E. coli* were then cultured and induced with 1 mM isopropyl-B-D-thiogalactopyranoside (Thermo Fisher Scientific, USA; Cat. No. R0392) for 16 h at 16°C in LB medium during the mid-exponential growth phase. Cells were harvested, sonicated, and protein was solubilized in PBS. Solubilized proteins were incubated with GST-Sepharose beads (GE Healthcare, USA; Cat. No. 17-5132-01) with rotation for 1 h at 4°C, collected by centrifugation, and washed five times with PBS. To determine whether the GST-WSV083 fusion protein binds to LvTcf, a pIEx-4-LvTcf-myc plasmid was transfected into High Five cells and harvested after 48 h, as mentioned above. The cell lysate was incubated with GST or GST-WSV083 fusion protein-bound beads with rotation at 4°C for 9 h, followed by five washes with PBS. Bound proteins were eluted in SDS loading buffer for Coomassie blue staining or western blotting.

### Dual-Luciferase Reporter Assay

Dual-luciferase reporter assays were performed in *Drosophila* S2 cells. Briefly, S2 cells were seeded into 48-well plates and cultured at 27°C in *Schneider’s Drosophila* Medium (Gibco, USA; Cat. No. R69007) supplemented with 10% fetal bovine serum (Gibco, USA; Cat. No. 16140071) and 1% Penicillin-Streptomycin (Gibco, USA; Cat. No. 15070063). Once cells grew to 60-80% confluence, transient transfection was performed. Using the FuGENE HD Transfection Reagent (Promega, USA; Cat. No. E2311), each well was transfected with 500 ng Firefly luciferase reporter plasmid (pGL3-Basic, pGL3-LvVago1-5, respectively), 500 ng of target protein expression plasmid (pIEx-LvTcf-Myc, pIEx-Lvβ-catenin-FLAG), and 5 ng pRL-OpIE2 Renilla luciferase plasmid (internal control). The transfection ratio of the three plasmids was 100:100:1. The cells were lysed 48 h after transfection, and luciferase activity was detected using the Dual-Luciferase^®^ Reporter Assay System (Promega, USA; Cat. No. E1910) on a GloMaxTM20/20 Luminometer (Promega, USA). Relative luciferase activity was calculated by normalizing Firefly luciferase activity to Renilla luciferase activity. Western blotting was performed to confirm protein expression. All experiments were repeated at least three times.

### Immunofluorescence Assay

Sf9 cells were transfected with the target plasmids and cultured for 24 h at 27°C in 35-mm dishes (Cellvis, USA; Cat. No. D35-20-1-N). The cells were washed twice with PBS, fixed with 4% paraformaldehyde at room temperature for 20 min, and were then permeabilized with 0.5% TritonX-100 (diluted in PBS) for 1 min. After washing three times, cells were blocked with 5% bovine serum albumin (diluted in TBST) for 1 h, followed by incubation for 1.5 h at room temperature or overnight (about 10 h) at 4°C with a mouse anti-myc antibody (Cell Signaling Technology, USA; Cat. No. 2276S; 1:250) and a rabbit anti-FLAG antibody (Abcam, USA; Cat. No. ab205606; 1:200) or a rabbit anti-V5 antibody (Thermo Fisher Scientific, USA; Cat. No. R961-25; 1:200). After washing with PBS four times, the cells were subsequently incubated with mixed FITC goat anti-mouse (Life technologies, USA, Cat. No. A11029; 1:500) and rhodamine goat anti-rabbit secondary antibodies (Life technologies, USA, Cat. No. R6394, 1:500) for 1 h at room temperature. The cells were washed with PBS five times before nuclei were stained with 0.5 μg/mL Hoechst (Beyotime, Nanjing, China; Cat. No. C1028) for 1 min. Finally, the cells were observed under a confocal laser-scanning microscope (Leica SP2).

### Calf Intestinal Alkaline Phosphatase (CIAP) Assay

LvTcf was co-expressed with EGFP, WSV083, or its mutants in High Five cells for 48 h. A spot of the cell lysate was used as input. The remaining proteins were immunoprecipitated with an anti-myc antibody and washed with lysis buffer three times as well as with phosphatase buffer (50 mM Tris-HCl, 0.1 mM EDTA, pH 8.5) three times. The bound beads in 30 μl phosphatase buffer were treated with CIAP (Thermo Fisher Scientific, USA; Cat. No. 18009-019) at 37°C for 1 h. The reaction was stopped with SDS loading buffer, and the beads were boiled for 5 min for western blotting.

### Ubiquitination Assay

Ubiquitination assays were performed in High Five cells. Expression plasmids, including ubiquitin, were co-transfected into cells. After 12 h, the cells were treated with 10 μM protease inhibitor MG132 (Merck, USA; Cat. No. 474790) for 24 h and harvested with Western and IP cell lysis buffer [20 mM Tris (pH7.5), 150 mM NaCl, 1% Triton X-100] (Beyotime, Nanjing, China; Cat. No. P0013). The supernatants were immunoprecipitated as described above. The input and immunoprecipitates were subjected to western blot analysis.

### Mass Spectrum Analysis

FLAG-LvTcf samples were collected from High Five cells co-expressing WSV083WT or WSV083DM. After SDS-PAGE analysis, FLAG-LvTcf bands were recovered for mass spectrometry analysis by Novogene (Beijing, China).

## Results

### Effects of LvTcf on WSSV Infection

We obtained full-length *LvTcf* from the *Litopenaeus vannamei* transcriptome analyzed in our laboratory (unpublished). The *LvTcf* gene was 2220 bp in length (GenBank accession No. MT241372), which included a 1671 bp ORF encoding 556-amino acid protein with a predicted molecular mass of 60.214 kDa. A catenin-binding domain, a conserved High Mobility group (HMG) domain, and a C-clamp domain were found at positions 1-56 aa, 288-358 aa, and 381-411 aa respectively (**Figures S1A, B**). Multiple sequence alignment suggested that the LvTcf HMG domain had several highly-conserved Ser/Thr/Lys phosphorylation sites (**Figure S1C**). Phylogenetic analysis revealed that LvTcf was evolutionarily associated with arthropods, including crustaceans and insects (**Figure S1D**).

**Figure 1:**
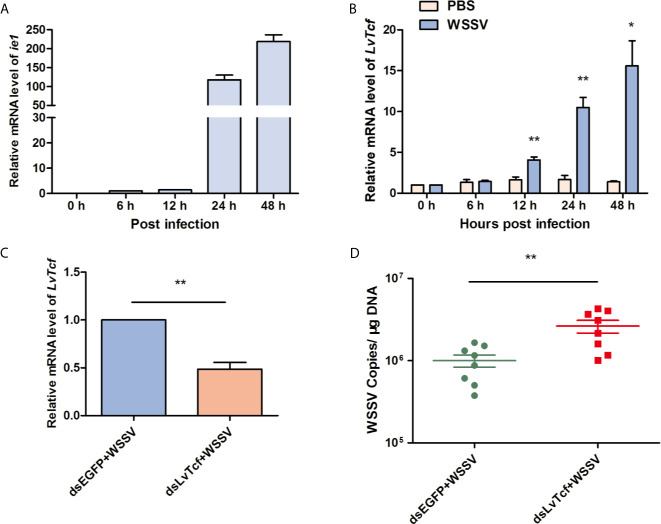
LvTcf played a protective role against WSSV infection. **(A, B)** LvTcf expression following WSSV infection. Expression of WSSV immediately early gene *ie1* **(A)** and *LvTcf* **(B)** after WSSV challenge, as determined using qRT-PCR. Hemocytes were collected at 0, 6, 12, 24, 48 h post-WSSV injection. *LvEF-1α* was used as internal control. **(C, D)** Effects of LvTcf on WSSV infection. **(C)** The expression level of *LvTcf* after RNAi. **(D)** Silencing of *LvTcf* facilitated WSSV proliferation. DsRNA-LvTcf and dsRNA-eGFP (control group) were injected into shrimp. After 24 h, the two groups were infected with 1×10^5^ WSSV virions. The number of WSSV copies in gills (n = 8) were assessed at 48 h post-infection by absolute q-PCR. *LvEF-1α* was used as internal control. All the experiments were performed in triplicate. The data were statistically analyzed *via* the student’s *t*-test (*p < 0.05, ** p < 0.01).

To investigate the relationship between LvTcf and WSSV infection, we performed time-course analysis of LvTcf expression in hemocytes. The expression of viral gene *ie1* was used as an index of WSSV infection level, increasing gradually, which suggested successful infection (**Figure 1A**). *LvTcf* expression was significantly upregulated approximately 6-fold at 24 hpi and 11-fold at 48 hpi (**Figure 1B**). These results indicated that WSSV infection positively regulated *LvTcf* expression. Further, we employed RNAi to knock down the expression of LvTcf in order to investigate its role during WSSV infection. Healthy shrimps were injected with dsLvTcf or dsEGFP (as control). Both groups were subsequently challenged with WSSV. 48 h after the injection of shrimp with WSSV, gill *LvTcf* was suppressed at the mRNA level compared to the control group (**Figure 1C**). The number WSSV copies in gills increased after LvTcf silencing (**Figure 1D**). Taken together, these results suggested that LvTcf plays a protective role against WSSV infection.

### LvTcf Promotes the Production of LvVago1 Independently of Lvβ-Catenin

**Figure 2:**
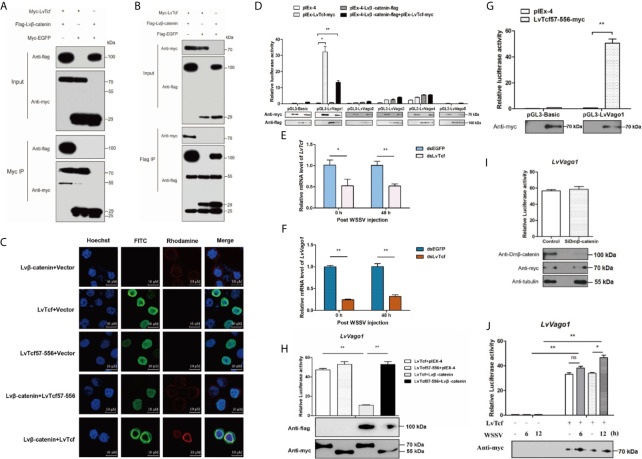
LvTcf was involved in regulating LvVago1 expression independently of Lvβ-catenin. **(A, B)** Co-IP analysis in High Five cells indicated that LvTcf interacted with Lvβ-catenin. Cell lysates were immunoprecipitated using rabbit anti-c-Myc agarose affinity Gel **(A)** or mouse anti-FLAG M2 affinity gel **(B)**. **(C)** Immunofluorescence analysis revealed that LvTcf colocalized with Lvβ-catenin. Cells were transfected with Myc-LvTcf, FLAG-Lvβ-catenin, or both. After 36 h, immunofluorescence analysis was performed with primary mouse anti-Myc, rabbit anti-FLAG, as well as secondary FITC anti-mouse and rhodamine anti-rabbit antibodies. Finally, the cells were observed under a confocal laser-scanning microscope. The scale bar = 10 μm. **(D–I)** LvTcf activated the *LvVago1* promoter independently of Lvβ-catenin. **(D)** The effects of Lvβ-catenin/LvTcf expression on the promoter activity of *LvVago1-5* were analyzed *via* a dual-luciferase reporter assay. **(E, F)** Silencing of *LvTcf* led to a decrease in *LvVago1* expression after WSSV infection. **(G, H)** The effect of LvTcf57-556/Lvβ-catenin on the promoter activity of *LvVago1* was analyzed *via* a dual-luciferase reporter assay. **(I)** Knockdown of Dmβ-catenin did not affect the LvTcf-induced activation of *LvVago1* promoter activity. **(J)** The effect of LvTcf on *LvVago1* upon WSSV stimulation. Data were analyzed *via* the student’s *t*-test (ns, No Significant, *p < 0.05, ** p < 0.01).

β-catenin and TCF usually form a complex in order to activate gene expression (37, 44, 45). As we predicted that the β-catenin-binding domain is located in the N-terminus of LvTcf, we sought to determine whether Lvβ-catenin could bind to LvTcf. Co-IP experiments indicated that Lvβ-catenin interacted with LvTcf in High Five cells (**Figures 2A, B**). Moreover, immunofluorescence analysis further revealed a strong colocalization of Lvβ-catenin with wild-type LvTcf, but not with its catenin-binding domain deletion mutation (LvTcf57-556) (**Figure 2C**). Lvβ-catenin expressed alone was mainly localized in the cytoplasm, while LvTcf, a nuclear protein, was present in the nucleus. When Lvβ-catenin and LvTcf were co-expressed in cells, Lvβ-catenin translocated into the nucleus and colocalized with LvTcf (**Figure 2C**).

According to previous reports, the β-catenin/TCF pathway facilitates interferon production following viral infection (30–32). Thus, we hypothesized that the Lvβ-catenin/LvTcf pathway could promote the expression of shrimp interferon-like protein Vago during WSSV infection. As five isoforms of Vago have been identified in shrimp to date (46, 47), we performed dual luciferase reporter gene assays in Drosophila S2 cells to confirm the Lvβ-catenin/LvTcf-mediated transcriptional regulation at Vago promoters. To our surprise, the results indicated that LvTcf, but not Lvβ-catenin, could significantly promote *LvVago1* promoter activity, and Lvβ-catenin/LvTcf together had a less effect (**Figures 2D**). Further, knockdown of LvTcf led to decreased *LvVago1* expression after WSSV infection (**Figures 2E, F**). We found that LvTcf57-556, a catenin-binding domain deletion mutant, could still activate the LvVago1 promoter (**Figure 2G**). This activation was not reduced under Lvβ-catenin co-expression (**Figure 2H**). To determine the influence of endogenous β-catenin in S2 cells, we silenced the expression of β-catenin *via* siRNA and detected the effect of LvTcf on the *LvVago1* promoter. The knockdown of endogenous Dmβ-catenin did not affect *LvVago1* promoter activity, which was regulated by LvTcf (**Figure 2I**).

Based on the above-described results, we suggest that LvTcf activates the expression of *LvVago1* independently of Lvβ-catenin. We further detected the regulatory activity of LvTcf on the *LvVago1* promoter upon WSSV stimulation. pGL3-LvVago1 was co-transfected with an empty vector or LvTcf expression plasmids in *Drosophila* S2 cells. WSSV was added at 36 and 42 h after transfection. After culturing for 48 h, the cells were lysed, and fluorescence activity was detected. WSSV stimulation increased *LvVago1* promoter activity, which was regulated by LvTcf (**Figure 2J**). Taken together, these results revealed that LvTcf could promote the expression of interferon-like protein LvVago1 independently of Lvβ-catenin, thus having a protective role against WSSV infection.

### LvTcf Interacts With WSV083

**Figure 3:**
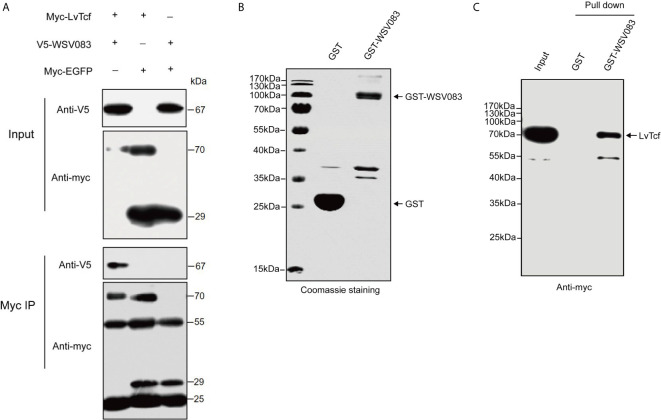
Interaction between LvTcf and WSV083. **(A)** Co-IP experiments indicated that LvTcf interacted with WSV083 in High Five cells. Myc co-IP was performed using a mouse anti-Myc antibody. Western blotting was performed using a mouse anti-Myc or anti-V5-HRP antibody. **(B, C)** GST pull-down analysis of the interaction between LvTcf and WSV083. **(B)** GST and GST-WSV083 were expressed in E. coli BL21 cells and purified using GST-Sepharose beads. Identical proteins were assessed by SDS-PAGE with Coomassie staining. **(C)** High Five cells transfected with Myc-LvTcf expression plasmids were lysed and incubated with GST Sepharose beads coupled with GST or GST-WSV083, respectively. The immunoprecipitates were analyzed via western blot using an anti-Myc antibody.

TCF is the main downstream nuclear transcription factor of the Wnt pathway and is involved in immune regulation. It is usually a target of diverse viruses (48). To reveal the regulatory effect of WSSV on host LvTcf, we sought to identify LvTcf-associated viral proteins. As key viral regulatory factors, WSSV immediate-early proteins play important roles in successful infection and replication. In addition, these proteins mainly localize to the host cell nucleus. Therefore, we selected WSSV immediate-early proteins with known functions to study their relationship with LvTcf. In High Five cells, viral immediate-early protein expression plasmids of WSV403, WSV249, WSV079, WSV083, WSV069, WSV100, and WSV056 were co-transfected with the LvTcf-myc expression plasmid. The results indicated that LvTcf may interact with WSV083 and WSV069 (**Figure S2**). Transient transfection and Co-IP further indicated that WSV083 interacted with LvTcf (**Figure 3A**). Moreover, WSV083-GST was expressed in *E. coli* BL21, while LvTcf-myc was expressed in High Hive cells due to its difficult expression in prokaryotic cells. A GST-pull-down was then performed, indicating that WSV083-GST, but not GST alone, could bind to LvTcf *in vitro* (**Figures 3B, C**). These results revealed that WSV083 interacted with LvTcf directly.

### WSV083 Phosphorylates LvTcf

**Figure 4:**
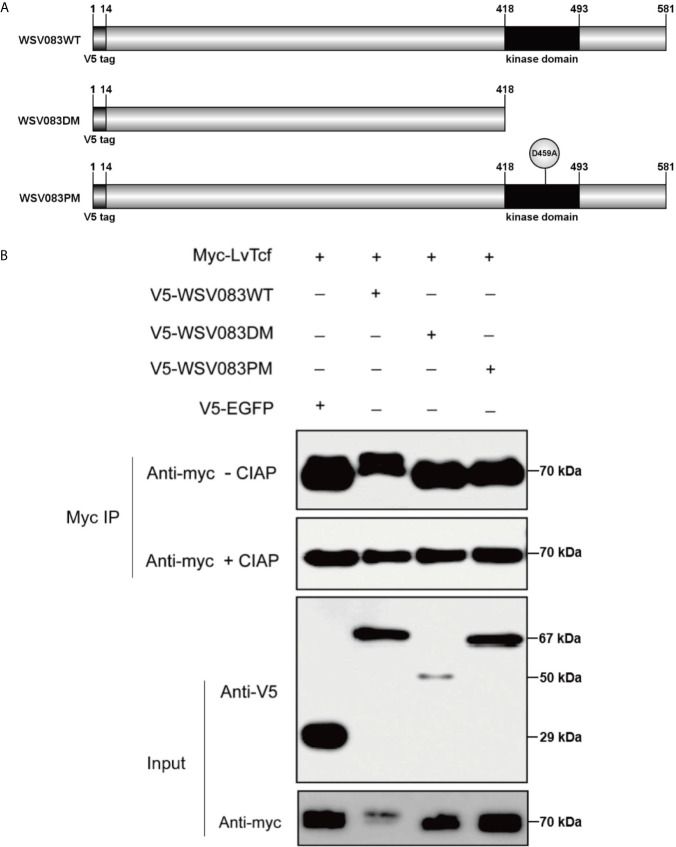
LvTcf phosphorylation by WSV083. **(A)** Schematic diagram of WSV083 and its kinase activity deficiency mutant expression vectors. **(B)** High Five cells were co-transfected with Myc-LvTcf and V5-EGFP, V5-WSV083WT, V5-WSV083DM, V5-WSV083PM, respectively. After 48 h, whole-cell extracts were immunoprecipitated with anti-Myc and protein A beads. Immunoprecipitates were then subjected to CIAP treatment and immunoblotting as indicated.

While exploring the interaction between LvTcf and WSV083, we found that LvTcf displayed an upshift in mobility on the SDS-PAGE gel when co-expressed with WSV083 (**Figure 3A**). As WSV083 is essentially a serine/threonine protein kinase, we hypothesized that this phenomenon was caused by the WSV083-mediated phosphorylation of LvTcf. At the same time, WSV083 kinase domain deletion mutant WSV083DM and kinase domain ATP-binding point mutant D459A WSV083PM expression plasmids were constructed (**Figure 4A**). We then co-expressed both LvTcf and WSV083 or its mutants in High Five cells. The LvTcf immunoprecipitated *via* anti-myc agarose was subjected to treatment with or without CIAP. The results revealed that LvTcf co-expressed with WSV083WT exhibited slower mobility through the gel compared to LvTcf co-expressed with the control vector. WSV083DM nor WSV083PM affected the mobility of LvTcf (**Figure 4B**). After CIAP treatment, the mobility difference of LvTcf disappeared (**Figure 4B**), indicating that WSV083 phosphorylated LvTcf.

### WSV083 Increases the Degradation of LvTcf Through the Ubiquitin-Proteasome Pathway

**Figure 5:**
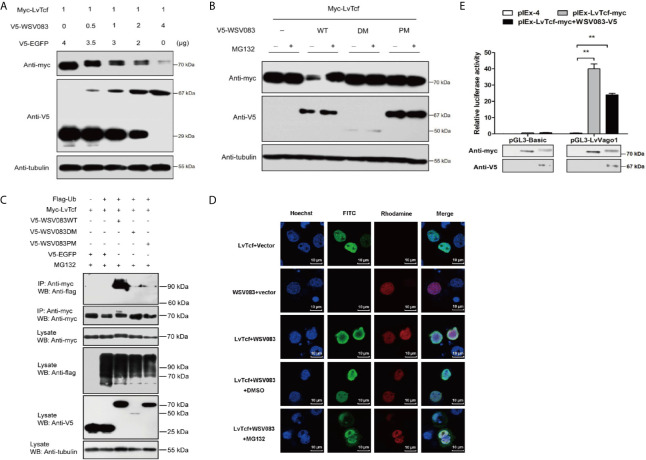
WSV083 promotes the degradation of LvTcf *via* the ubiquitin-proteasome pathway. **(A)** High Five cells were transfected with Myc-LvTcf and increasing doses of V5-WSV083 expression plasmid (0, 0.5, 1, 2, and 4 μg), as indicated. After 48 h, the cells were lysed and analyzed by western blotting. **(B)** High Five cells were co-transfected with Myc-LvTcf, pIEx-4, V5-WSV083WT, or its mutants. After 24 h, the cells were treated with or without 10 μM MG132 as indicated. Anti-Myc, anti-V5, and anti-tubulin antibodies were used for western blotting. **(C)** WSV083 promoted the polyubiquitination of LvTcf. High Five cells were co-transfected with Myc-LvTcf, FLAG-Ub, and V5-WSV083, as indicated. After 24 h, the cells were treated with 10 μM MG132 for 12 h. Whole-cell extracts were immunoprecipitated with anti-Myc and protein A beads followed by western blotting with anti-FLAG. **(D)** WSV083-phosphorylated LvTcf was transferred into the cytoplasm for degradation. Cells were transfected with Myc-LvTcf, V5-WSV083, or both. After 24 h, cells co-transfected with Myc-LvTcf and V5-WSV083 were treated with MG132 or DMSO for 18 h. Immunofluorescence experiments were performed with a primary mouse anti-Myc, rabbit anti-V5 as well as secondary FITC anti-mouse and rhodamine anti-rabbit antibodies. Finally, the cells were observed under a confocal laser-scanning microscope. Scale bar = 10 μm. **(E)** Dual-luciferase reporter assays indicated that WSV083 inhibited the effect of LvTcf on the *LvVago1* promoter. Data were analyzed *via* the student’s *t*-test (**p < 0.01).

In addition, we found that LvTcf protein levels were reduced in the presence of WSV083WT (**Figures 3A**, **4B**). To validate this observation, we transfected High Five cells with equal amounts of LvTcf plasmid and increasing amounts of WSV083WT plasmid, while decreasing amounts of EGFP plasmid were added to maintain the same amount of transfection. Western blotting results indicated that WSV083 decreased the level of LvTcf in a dose-dependent manner (**Figure 5A**). To further confirm whether WSV083 promoted the degradation of LvTcf through the proteasome pathway after its phosphorylation, we co-transfected LvTcf with pIEx-4, WSV083WT, WSV083DM, and WSV083PM expression plasmids into High Five cells. The proteasome inhibitor MG132 was used to block protein degradation. MG132 completely inhibited LvTcf degradation by WSV083WT, while the empty vector, WSV083DM, or WSV083PM, had no effect on LvTcf (**Figure 5B**). This suggests that phosphorylation of LvTcf by functional WSV083 is necessary for its proteasome-mediated degradation.

Since protein degradation is usually related to ubiquitination, we further determined whether LvTcf underwent ubiquitylation as a result of WSV083-mediated phosphorylation. LvTcf was co-transfected with EGFP, WSV083, and ubiquitin into High Five cells for intracellular ubiquitination assays. In the presence of WSV083WT, which possesses kinase activity, a polyUb-LvTcf smear of greater molecular mass was observed compared to the control group (**Figure 5C**), indicating that WSV083 promoted the ubiquitylation of LvTcf. Moreover, immunofluorescence analysis indicated that LvTcf colocalized with WSV083, and the latter could change the subcellular localization of LvTcf upon addition of MG132 (**Figure 5D**). We believe that WSV083-phosphorylated LvTcf was transferred to the cytoplasm for degradation. The current results suggested that WSV083 increased the degradation of LvTcf *via* the ubiquitin-proteasome pathway. In addition, WSV083 inhibited the LvTcf-mediated activity of the *LvVago1* promoter (**Figure 5E**). While our data cannot rule out an indirect mechanism where WSV083 regulates a kinase that in turn directly phosphorylates LvTCF, we favor a model where WSV083 phosphorylates LvTCF directly, on multiple residues that remain to be identified, leading to its degradation by the ubiquitin-proteasome pathway.

### Identification of WSV083-Targeted LvTcf Phosphorylation Sites

**Figure 6:**
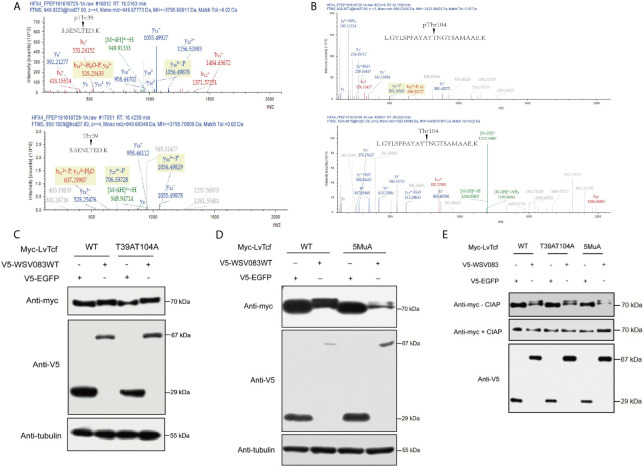
Identification of WSV083 phosphorylation sites on LvTcf. **(A, B)** Mass spectrometry analysis of LvTcf immunoprecipitates revealed that Thr39 and Thr104 in LvTcf were phosphorylation sites targeted by WSV083. **(A)** or **(B)** upper panel: Spectrum analysis of LvTcf immunoprecipitated from cells co-expressing WSV083WT; lower panel: from cells co-expressing WSV083DM. **(C, D)** Myc-LvTcfT-39AT104A or Myc-LvTcf5MuA (T39A/T104A/T311A/S315A/S356A) still displayed their mobility shift when co-expressed with WSV083WT. Myc-LvTcfWT, Myc-LvTcfT39AT104A **(C)**, Myc-LvTcf5MuA **(D)** were co-expressed with V5-EGFP or V5-WSV083WT in High Five cells. After 36 h, cell lysates were subjected to western blotting, as indicated. **(E)** CIAP abolished the mobility shift of LvTcf mutants when co-expressed with WSV083WT. Myc-LvTcfWT, Myc-LvTcfT39AT104A, or Myc-LvTcf5MuA were co-expressed with V5-EGFP or V5-WSV083WT, respectively. At 36 h post-transfection, cell lysates were subjected to immunoprecipitation, CIAP treatment, and immunoblotting, as indicated.

Based on the results described above, we speculated that an E3 ligase might recognize the specific LvTcf residues that were phosphorylated by WSV083. To locate these, we employed mass spectrometry in LvTcf samples gathered from High Five cells co-expressing LvTcf with WSV083WT or WSV083DM (**Figure S3**). The Thr39 and Thr104 LvTcf residues were found to be phosphorylated in cells co-transfected with WSV083WT, but not WSV083DM (**Figures 6A, B**). We therefore suggest that WSV083 phosphorylates LvTcf at Thr39 and Thr104. However, the LvTcfT39AT104A mutant (LvTcf2MuA) retained a mobility shift in SDS-PAGE gel when co-expressed with WSV083WT (**Figure 6C**). LvTcf might therefore contain other residues that are phosphorylated by WSV083, in addition to Thr39 and Thr104. Thus, we continued to substitute some potentially conserved phosphorylated serine or threonine residues (Thr311, Ser315, and Ser356) with alanine. When T39, T104, Thr311, Ser315 and Ser356 (LvTcf5MuA) were simultaneously mutated, a mobility shift still occurred (**Figure 6D**). CIAP treatment abolished the mobility difference of LvTcf mutants (**Figure 6E**). These results indicated that a series of serine or threonine sites of LvTcf, including T39 and T104, could be phosphorylated by WSV083.

## Discussion

TCF, the key transcription factor of the Wnt signaling pathway, drives the development of lymphocytes (T cells, B cells), NK cells, and innate lymphoid cells (49, 50). Further, TCF-1 is essential for both the initiation of TFH differentiation and the effector function of differentiated TFH during acute viral infection by promoting the expression of Bcl-6 (28). In addition, the β-catenin/TCF signaling pathway functions in innate defense mechanisms to defend against diverse pathogens *via* phagocytosis, autophagy, reactive oxygen species production, and antimicrobial peptide production (31). Thus, TCF plays a critical role in innate and adaptive immune responses. Various studies have confirmed the involvement of Wnt signaling in WSSV infection (33–35, 51–54). However, little is known regarding the relationship between shrimp Tcf and WSSV. In the current study, we demonstrated for the first time that LvTcf exerts antiviral effects by promoting the production of interferon-like protein LvVago1 independently of Lvβ-catenin. Further, this antiviral effect was suppressed by WSSV protein kinase WSV083.

WSSV triggered expression changes in diverse host molecules, including LvTcf. As shown in **Figure 1**, LvTcf mRNA was significantly increased following WSSV infection (**Figures 1A, B**), indicative of a close relationship between LvTcf and WSSV infection. RNAi experiments revealed that LvTcf knockdown resulted in greater WSSV proliferation (**Figures 1C, D**), suggesting that the former plays a protective role against WSSV infection. A recent research found that two isoforms of LvPangolin, LvPangolin1 (LvTcf in this work) and LvPangolin2, had antiviral properties against WSSV, which is compatible with our findings (55).

Wnt/β-catenin/TCF signaling pathway-mediated responses to bacterial and viral infections have been the subject of extensive study, highlighting the cascade’s roles in orchestrating phagocytosis, antimicrobial or interferon defense, as well as inflammatory cytokine production (56–58). In the current study, we sought to explore the LvTcf-mediated regulation of downstream immune-related genes in shrimp. Interferons, a family of immune proteins which spearhead antiviral defense, are regulated by β-catenin/TCF in an interferon regulatory factor-independent manner (59). There are five interferon-like proteins in shrimp. Among them, the interferon regulatory factor-mediated antiviral effects of LvVago1, LvVago4, and LvVago5, have been previously characterized (47, 60). Sequence analysis revealed that LvTcf harbors several possible binding sites in the promoters of LvVago-encoding genes. LvTcf promoted the activity of the *LvVago1* promoter during WSSV infection (**Figures 2D–F, H, J**). In a recent study, β-catenin was shown to activate gene expression by binding to other transcription factors such as FOXO4 without TCF (61). We found that LvTcf initiated the production of LvVago1 independently Lvβ-catenin even though they could interact with each other. Moreover, cooperation with Lvβ-catenin reduced the effect of LvTcf on *LvVago1* promoter activity. It is suggested that LvTcf acts by itself or cooperates with other transcription factors to activate LvVago1 expression, whereas the Lvβ-catenin/LvTcf complex might upregulate the transcription of other shrimp target genes. Taken together, our study clarified one of the mechanisms through which LvTcf defends against WSSV infection.

Host immune molecules are often targeted by viruses. The regulation of post-translational modifications, especially the ubiquitination of host immune molecules, is an effective means of immune evasion (20, 62). For instance, IpaH9.8, an E3 ubiquitin ligase of Shigella flexneri, could target human GBP-1 to induce its ubiquitination and degradation, thus suppressing host defense (63). Kaposi’s sarcoma-associated herpesvirus encodes an E3 ligase RTA that suppresses innate immunity *via* the ubiquitin-mediated degradation of MyD88 (64). As pivotal nuclear co-activators of the Wnt signaling pathway, TCFs are regulated by various factors. LEF1, TCF7L1, and TCF7L2 are phosphorylated by HIPK2 at their HMG box domain (65). These are also phosphorylated by NLK, as the first step for ubiquitylation by NARF, and are subsequently degraded *via* the proteasome (66). Various reports have indicated that SUMOylation and acetylation of TCFs regulate their subcellular localization and transcriptional activity (67, 68). In the present study, we discovered that LvTcf was modulated by the viral Ser/Thr protein kinase WSV083. WSV083 caused the phosphorylation of LvTcf and delayed its mobility in SDS-PAGE due to changes in molecular weight and isoelectric point (**Figure 4B**). WSV083-mediated LvTcf phosphorylation could impact the latter’s protein stability (**Figures 5A–C**). Phosphorylated LvTcf was transferred into the cytoplasm for degradation *via* the ubiquitin-proteasome pathway (**Figure 5D**). Some E3 ligases mediate the degradation of target proteins necessary for the recognition of specific phosphorylated amino acid residues (69–71). We speculated that an E3 ligase might recognize the LvTcf residues phosphorylated by WSV083 prior to its ubiquitination. However, mass spectrometry analysis did not identify all LvTcf residues targeted by WSV083 (**Figure 6**). In addition, although over-expression of WSV083 downregulated LvTcf protein and inhibited its ability to activate *Lv*Vago1 promoter (**Figure 5E**), this did not contradict the findings in **Figure 2J** that WSSV infection induced LvTcf activation of the *Lv*Vago1. In contrast to WSV083 overexpression, the amount of WSV083 in S2 cells was insufficient to cause LvTcf breakdown in a WSSV-stimulated manner. S2 cells, on the other hand, were capable of receiving and responding to WSSV infection. In response to WSSV stimulation, LvTcf in S2 cells was likely to upregulate the activity of the *Lv*Vago1 promoter.

**Figure 7:**
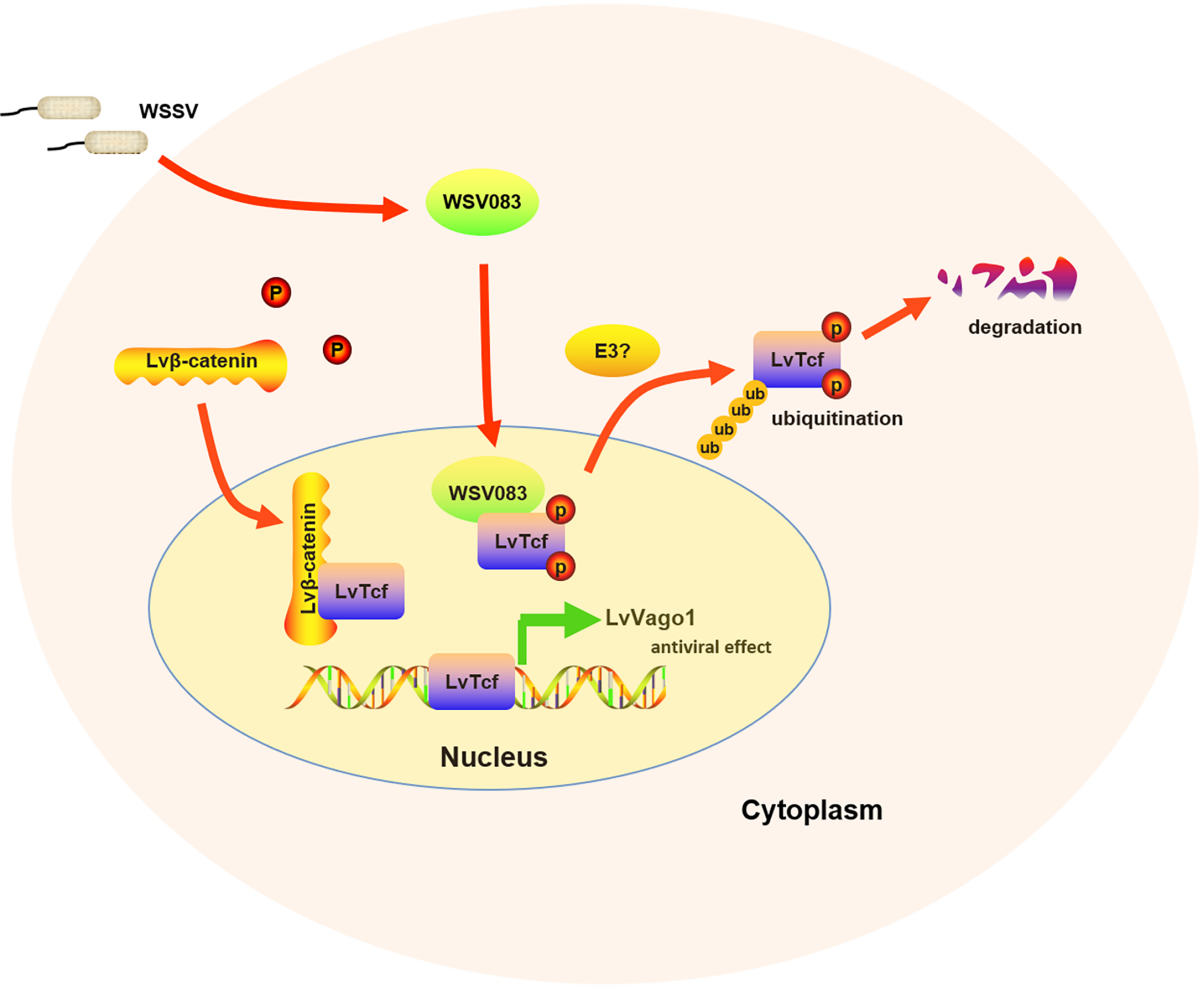
Model of the relationship between LvTcf and WSSV infection. WSSV infection (pathogenic stress) induced the LvTcf-driven production of interferon-like protein LvVago1 independently of Lvβ-catenin, as an antiviral defense mechanism. However, WSSV encodes a serine/threonine protein kinase, WSV083, which phosphorylates LvTcf. Phosphorylated LvTcf is translocated to the cytoplasm, recognized by an E3 ligase, and degraded *via* the ubiquitin-proteasome pathway.

In summary, our work showed that LvTcf promoted the expression of shrimp interferon-like protein Vago1 following WSSV infection, whereas WSSV protein kinase WSV083 could directly bind to and phosphorylate LvTcf, promoting its degradation *via* the ubiquitin-proteasome pathway (**Figure 7**). The current findings provide new insight into the response against WSSV, thus having implications for disease control.

## Data Availability Statement

The datasets presented in this study can be found in online repositories. The names of the repository/repositories and accession number(s) can be found in the article/**Supplementary Material**.

## Author Contributions

CW, LR, and HS designed the experiments. CW, WL, LL, and SL performed the experiments and data analysis. CW and LR were responsible for writing, finalizing, and submitting the manuscript. All the authors discussed the results. All authors contributed to the article and approved the submitted version.

## Funding

This work was funded by Natural Science Foundation of Fujian Province of China (2020J06031), National Natural Science Foundation of China (No. 31972816) and China Agriculture Research System of MOF and MARA (CARS-48).

## Conflict of Interest

The authors declare that the research was conducted in the absence of any commercial or financial relationships that could be construed as a potential conflict of interest.

## Publisher’s Note

All claims expressed in this article are solely those of the authors and do not necessarily represent those of their affiliated organizations, or those of the publisher, the editors and the reviewers. Any product that may be evaluated in this article, or claim that may be made by its manufacturer, is not guaranteed or endorsed by the publisher.
