# Supplementary material for: Phosphorylation of Shrimp Tcf by a Viral Protein Kinase WSV083 Suppresses Its Antiviral Effect
Source: Front Immunol. 2021 Aug 2;12:698697. doi: 10.3389/fimmu.2021.698697 (PMC8365339; doi:10.3389/fimmu.2021.698697)
Supplement: Supplementary file 1 [file DataSheet_1.docx]

Supplementary Material

## Supplementary Figures

**
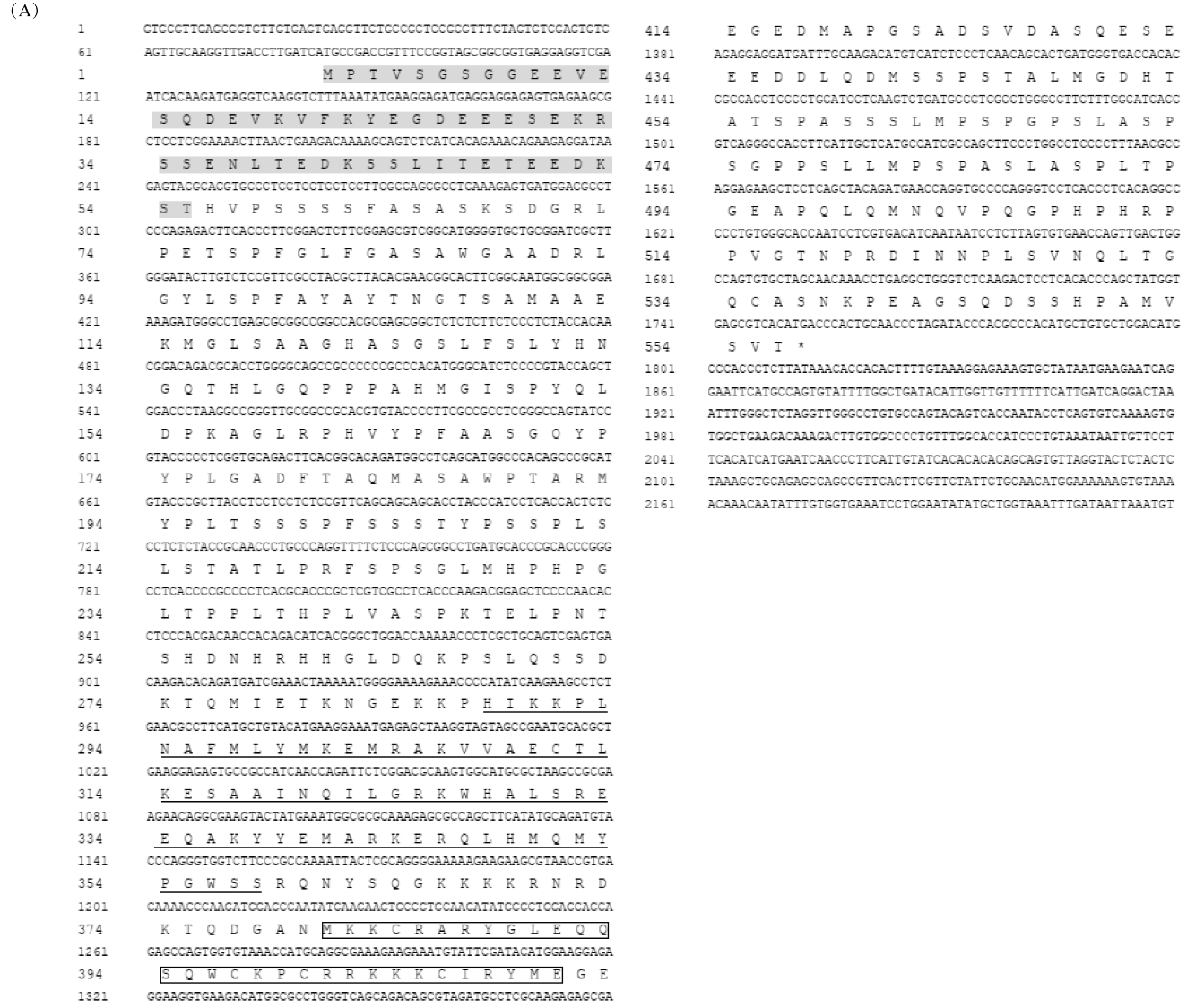
**

**
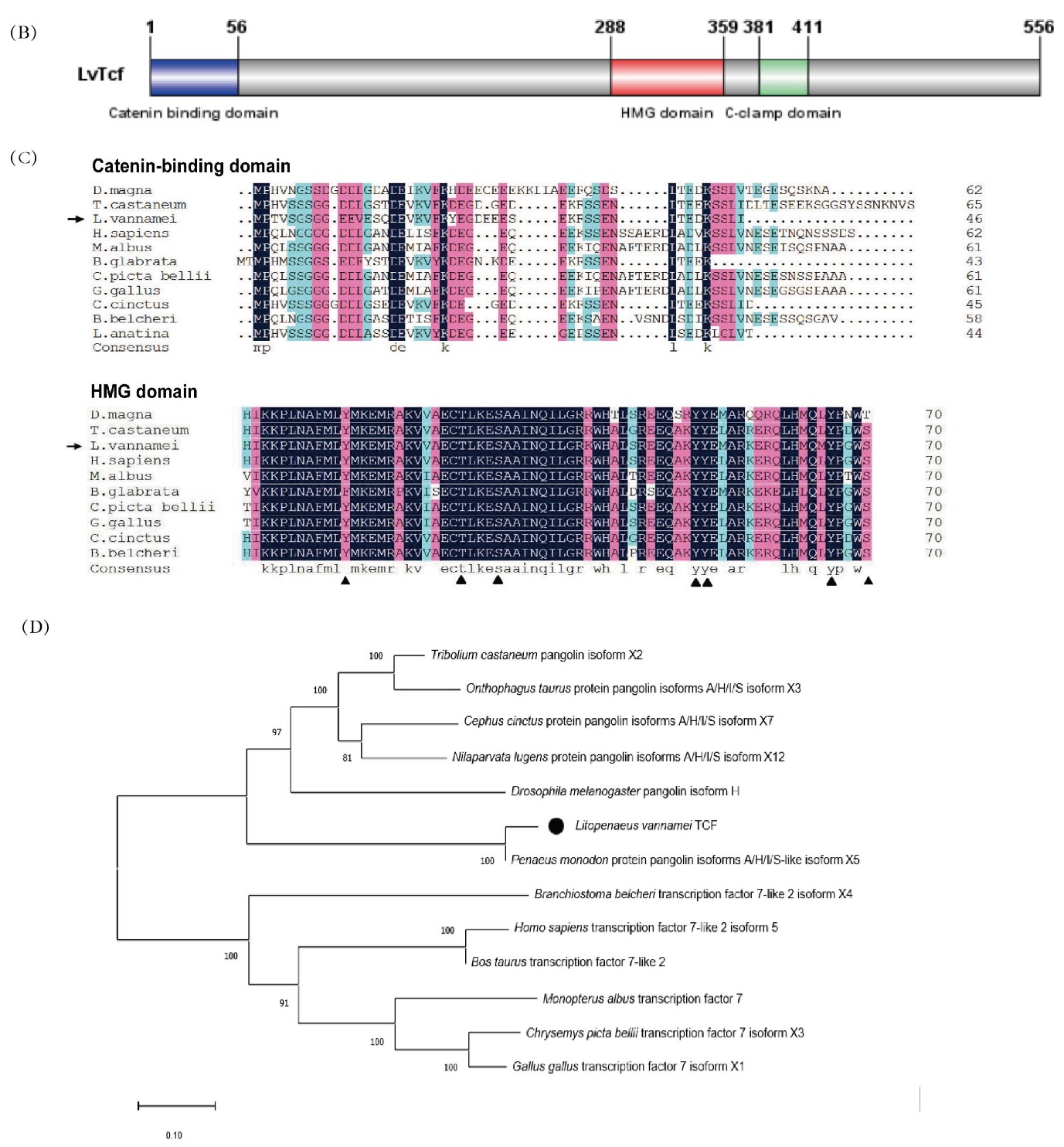
**

**Figure S1. Sequence analysis of LvTcf, related to Figure 1.**

**(A)** Nucleotide and deduced amino acid sequence of LvTcf. Catenin-binding domain (residues 1-56) was indicated with grey shadow. The sequence of HMG domain was underlined (residues 288-358) **(B)** Architecture of LvTcf. **(C)** Multiple sequence alignment among Tcf of *L. vannamei* and other species. **(D)** Phylogenetic analysis based on the amino-acid sequences of Tcfs. Protein sequences of different species were listed below: *L. vannamei* Tcf (MT241372), *Tribolium castaneum* pangolin isoform X2 (XP_008191150.1), *Cephus cinctus* protein pangolin, isoforms A/H/I/S isoform X7 (XP_015597923.1), *Drosophila melanogaster* pangolin, isoform H (NP_001014685.1), *Branchiostoma belcheri* transcription factor 7-like 2 isoform X4 (XP_019644519.1), *Chrysemys picta bellii* transcription factor 7 isoform X3 ([XP_005309518.1](https://www.ncbi.nlm.nih.gov/protein/XP_005309518.1?report=genbank&log$=prottop&blast_rank=1&RID=0YFAZ8T6014)), *Gallus gallus* transcription factor 7 isoform X1 (XP_015149481.1), *Monopterus albus* transcription factor 7 (XP_020467632.1), *Homo sapiens* transcription factor 7-like 2 isoform 5 (NP_001139757.1), *Penaeus monodon* protein pangolin, isoforms A/H/I/S-like isoform X5 (XP_037783988.1), *Nilaparvata lugens* protein pangolin, isoforms A/H/I/S isoform X12 ( XP_039276382.1), *Bos taurus* transcription factor 7-like 2 (NP_001137211.1), *Onthophagus taurus* protein pangolin, isoforms A/H/I/S isoform X3 (XP_022914178).

**
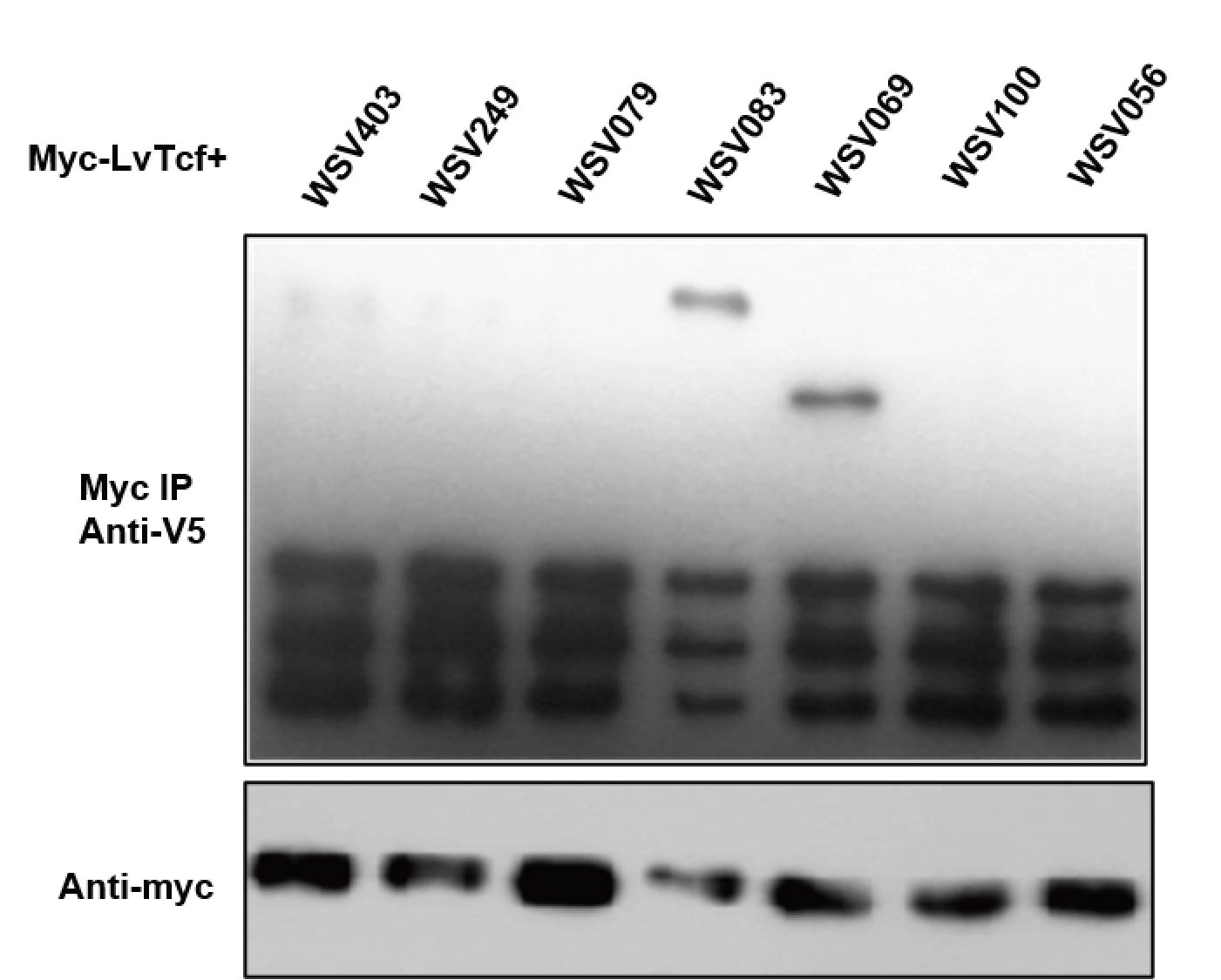
**

**Figure S2. Screening of WSSV proteins that interact with LvTcf, related to Figure 3.**

WSSV immediate-early genes encoding WSV403, WSV249, WSV079, WSV083, WSV069, WSV100, and WSV056 were cloned into the pIEx-4 plasmid and co-expressed with a LvTcf expression plasmid in High Five cells. After 48 h, cell lysates were used for Co-IP experiment and western blotting.

**
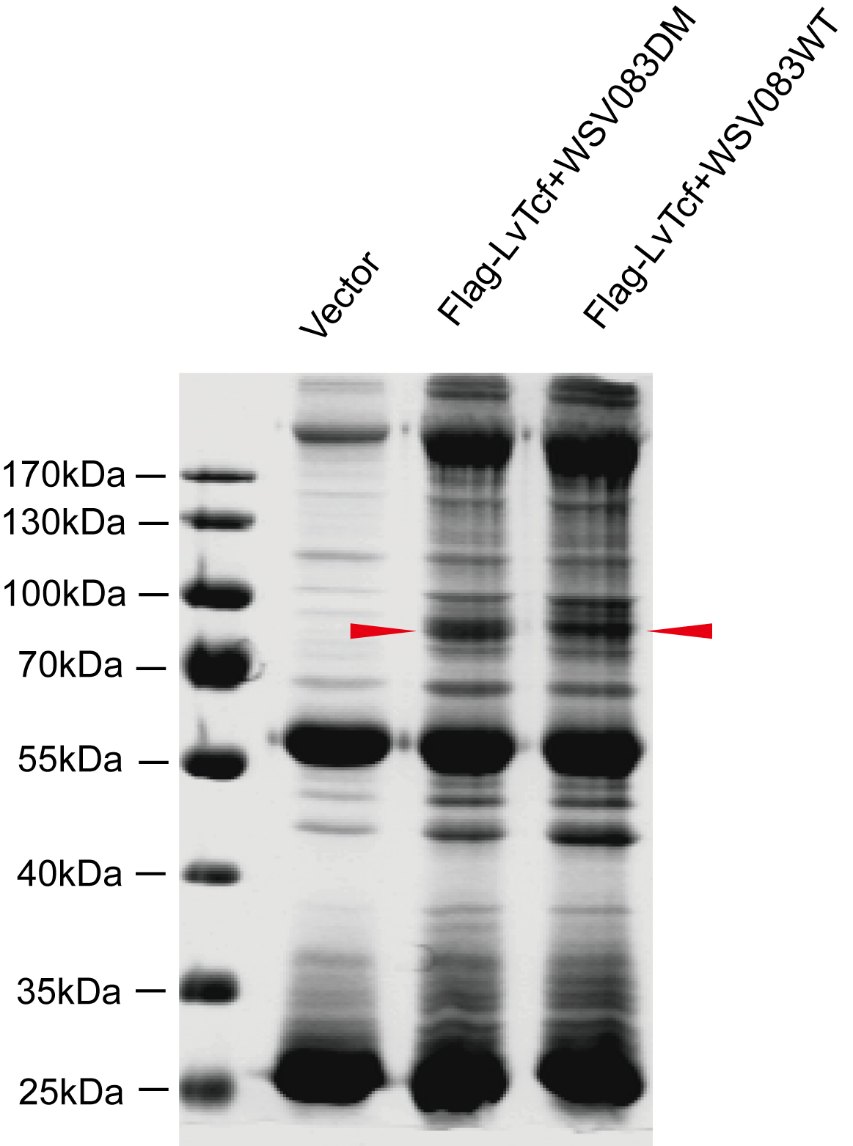
**

**Figure S3. The purification of LvTcf expressed in High Five cells, related to Figure 6.**

FLAG-LvTcf co-expressed with pIEx-4, WSV083WT or WSV083DM in High Five cells. After 48 h, cell lysates were immunoprecipitated by an Anti-FLAG M2 Affinity Gel. The purified products were analyzed using SDS-PAGE and stained with Coomassie blue. The target bands (as indicated) were the cut for mass spectrometry analysis.
