# Supplementary material for: Phosphorylation of Shrimp Tcf by a Viral Protein Kinase WSV083 Suppresses Its Antiviral Effect
Source: Front Immunol. 2021 Aug 2;12:698697. doi: 10.3389/fimmu.2021.698697 (PMC8365339; doi:10.3389/fimmu.2021.698697)
Supplement: Supplementary file 2 [file Table_1.docx]

**Table S1 The primers used in this study.**

| **Names** | **Sequence (5’-3’)** |
| --- | --- |
| **Sequence validation** | |
| LvTcf-F | GTGCGTTGAGCGGTGTTGTGAGTG |
| LvTcf-R | AGAAAAGCAAGACTGTCTCCATACT |
| **qRT-PCR** | |
| LvEF-1α-qF | GTATTGGAACAGTGCCCGTG |
| LvEF-1α-qR | ACCAGGGACAGCCTCAGTAAGA |
| ie1-qF | GCACAACAACAGACCCTACCC |
| ie1-qR | GAAATACGACATAGCACCTCCAC |
| LvTcf-qF | AGGTGCGTCTGTCCGTTG |
| LvTcf-qR | TCGCTTGGGATACTTGTCTC |
| LvVago1-qF | AGCCTCGTCTTCGCACAACAG |
| LvVago1-qR | CGGAAGTTTGTTGACGAGAAG |
| WSSV genome DNA-F | TGAAAGCAGACGCTCCCTTAC |
| WSSV genome DNA-R | CACCCACAGCGGCTCTTG |
| **RNAi** | |
| dsRNA-eGFP-F | GTGCCCATCCTGGTCGAGCT |
| dsRNA-T7-eGFP-R | GGATCCTAATACGACTCACTATAGGTGCACGCTGCCGTCCTCGAT |
| dsRNA-eGFP-R | TGCACGCTGCCGTCCTCGAT |
| dsRNA-T7-eGFP-F | GGATCCTAATACGACTCACTATAGGGTGCCCATCCTGGTCGAGCT |
| dsRNA-LvTcf-F | CCACGACAACCACAGACATCACG |
| dsRNA-LvTcf-T7-R | GGATCCTAATACGACTCACTATAGGTCTTGCGAGGCATCTACGCTGTC |
| dsRNA-LvTcf-T7-F | GGATCCTAATACGACTCACTATAGGCCACGACAACCACAGACATCACG |
| dsRNA-LvTcf-R | TCTTGCGAGGCATCTACGCTGTC |
| **Dual-luciferase reporter assay** | |
| pGL3-LvVago1P-F | CGGGGTACCATGCACGTCCACACACAAACTCAC |
| pGL3-LvVago1P-R | CCGCTCGAGTTCCCAAGCAAGCAATCAGCAAG |
| pGL3-LvVago2P-F | CGGGGTACCTATAGGGCACGCGTGGTCGACG |
| pGL3-LvVago2P-R | GGAAGATCTCAGGGAAGTCTTTGTGAACCTCTG |
| pGL3-LvVago3P-F | CGGGGTACCAGTGTTTGCCCCGTTCCCGTGTGC |
| pGL3-LvVago3P-R | GGAAGATCTCATAGTGACTTGTTGCTGGTGGTGC |
| pGL3-LvVago4P-F | CGGGGTACCGGCAACGCAAGAGATCCCTAATAT |
| pGL3-LvVago4P-R | CCGCTCGAGACTTGCGCGACGCTAAAACCCATT |
| pGL3-LvVago5P-F | CGGGGTACCTTAGCCCTTTTACCAATTAATCATC |
| pGL3-LvVago5P-R | GGAAGATCTCCATCCTTATACCGGGTCCTCCTCC |
| **Protein expression** | |
| pGEX-WSV083-F | CGCGGATCCATGGGGGGACCCACTGTAATTAC |
| pGEX-WSV083-R | CCGCTCGAGTTCATTTCTTTACTTTAAACAGGTTC |
| pIEx-V5-EGFP-F | CGCGGATCCCGGTAAGCCTATCCCTAACCCTCTCCTCGGTCTCGATTCTACGATGGTGAGCAAGGGCGAGGAG |
| pIEx-Myc-EGFP-F | CGCGGATCCCGAGCAGAAACTCATCTCTGAAGAGGATCTGATGGTGAGCAAGGGCGAGGAG |
| pIEx-FLAG-EGFP-F | CGCGGATCCCGATTACAAGGATGACGACGATAAGATGGTGAGCAAGGGCGAGGAG |
| pIEx-EGFP-R | CCCAAGCTTTTACTTGTACAGCTCGTCCATGC |
| pIEx-FLAG-ub-F | CATGCCATGGCAGATTACAAGGATGACGACGATAAGATGGGATCCATGCAGATCTTCG |
| pIEx-ub-R | CCCAAGCTTTTAATAAGATCTGGTACCGAGC |
| pIEx-Myc-LvTcf-F | CGCGGATCCCGAGCAGAAACTCATCTCTGAAGAGGATCTGATGCCGACCGTTTCCGGTAGCG |
| pIEX-Myc-LvTcf57-556-F | CGCGGATCCGAGCAGAAACTCATCTCTGAAGAGGATCTGCGTGCCCTCCTCCTCCTCCTTCG |
| pIEx-LvTcf-R | CCCAAGCTTTCATGTGACGCTCACCATAGCTG |
| pIEx-FLAG-LvTcf-F | CGCGGATCCCGATTACAAGGATGACGACGATAAGATGCCGACCGTTTCCGGTAGCG |
| pIEx-LvTcf-R | CCCAAGCTTTCATGTGACGCTCACCATAGCTG |
| pIEx-FLAG-Lvβ-catenin-F | CGAGCTCGATTACAAGGATGACGACGATAAGATGAGTTATCAGATGCCTCAGCAAG |
| pIEx-Lvβ-catenin-R | CCCAAGCTTTTACAGGTCCGTGTCATACCAGG |
| pIEx-V5-WSV083WT-F | CGCGGATCCCGGTAAGCCTATCCCTAACCCTCTCCTCGGTCTCGATTCTACGATGGGGGGACCCACTGTAATTAC |
| pIEx-WSV083WT-R | CCCAAGCTTTCATTTCTTTACTTTAAACAGGTTC |
| pIEx-V5-WSV083DM-F | CGCGGATCCCGGTAAGCCTATCCCTAACCCTCTCCTCGGTCTCGATTCTACGATGGGGGGACCCACTGTAATTAC |
| pIEx-WSV083DM-R | CCCAAGCTTTTAAGGCATGTTCACCTTTAG |
| pIEx-V5-WSV083PM-F1 | CGCGGATCCCGGTAAGCCTATCCCTAACCCTCTCCTCGGTCTCGATTCTACGATGGGGGGACCCACTGTAATTAC |
| pIEx-WSV083PM-R1 | GGTATGAGAGTCCCAAGGCAATCATT |
| pIEx-WSV083PM-F2 | AATGATTGCCTTGGGACTCTCATACC |
| pIEx-WSV083PM-R2 | CCCAAGCTTTCATTTCTTTACTTTAAACAGGTTC |
| pIEx-V5-WSV056-F | CGCGGATCCCGGTAAGCCTATCCCTAACCCTCTCCTCGGTCTCGATTCTACGATGGCCTCAGTCTTTGAAGACCCTG |
| pIEx-WSV056-R | CCCAAGCTTTTATTGTACCAAAAACTCAGAAATC |
| pIEx-V5-WSV079- F | CGCGGATCCCGGTAAGCCTATCCCTAACCCTCTCCTCGGTCTCGATTCTACGATGGCTGAAACCGTCGCCGTTGATG |
| pIEx-WSV079-R | CCGCTCGAGTTATAGATTGGGCAGGGAAGATATT |
| pIEx-V5-WSV100-F | CGAGCTCGGTAAGCCTATCCCTAACCCTCTCCTCGGTCTCGATTCTACGATGTCCTCGACGACCTCTCCATCG |
| pIEx-WSV100-R | CCGCTCGAGTCAGTCATCCATGTCGTTGCGAAC |
| pIEx-V5-WSV249-F | CGCGGATCCCGGTAAGCCTATCCCTAACCCTCTCCTCGGTCTCGATTCTACGATGGCAGCAGCAGCAGTCTCAGG |
| pIEx-WSV249-R | CCGCTCGAGTTATGTCTTGAACGAGAAATTG |
| pIEx-V5-WSV403-F | CGCGGATCCCGGTAAGCCTATCCCTAACCCTCTCCTCGGTCTCGATTCTACGATGGTTGCTTCAACTCCGTGTCC |
| pIEx-WSV403-R | CCGCTCGAGTTATTCGTTTAACACACACACAC |
| **Point mutations** | |
| pIEx-LvTcfT39A-F | CTCGGAAAACTTAgcaGAAGACAAAAGCAGTCTCATCACAG |
| pIEx-LvTcfT39A-R | CtgcTAAGTTTTCCGAGGAGCGCTTCTCACTC |
| pIEx-LvTcfT104A-F | TACGCTTACgcaAACGGCACTTCGGCAATG |
| pIEx-LvTcfT104A-R | CCGTTtgcGTAAGCGTAGGCGAACGGAG |
| pIEx-LvTcfT311S315A-F | GAATGCgcaCTGAAGGAGgcaGCCGCCATCAA |
| pIEx-LvTcfT311S315A-R | TCCTTCAGtgcGCATTCGGCTACTACCTTAGCTCT |
| pIEx-LvTcfS356A-F | GgcaTCCCGCCAAAATTACTCGCAGGGGAAAA |
| pIEx-LvTcfS356A-R | AATTTTGGCGGGAtgcCCACCCTGGGTACATCTGCA |
